# 9-Phenyl-4,5-diaza-9*H*-fluoren-9-ol monohydrate

**DOI:** 10.1107/S1600536812021940

**Published:** 2012-05-19

**Authors:** Guo-Jie Yin, Gang-Bin Yang, Shi-Min Wang

**Affiliations:** a Department of Environment Engineering and Chemistry, Luoyang Institute of Science and Technology, 471023 Luoyang, People’s Republic of China; b Chemistry Department, Zhengzhou University, 450052 Zhengzhou, People’s Republic of China

## Abstract

The title compound, C_17_H_12_N_2_O·H_2_O, was synthesized by the reaction of 4,5-diaza­fluoren-9-one with a Grignard reagent in ether (the reaction mixture being hydrolysed with saturated NH_4_Cl solution), and crystallizes with two organic mol­ecules and two water mol­ecules in the asymmetric unit. The 4,5-diaza­fluorene fragment is approximately planar, with r.m.s. deviations of 0.0448 and 0.0198 Å in the two mol­ecules. The dihedral angles between the 4,5-diaza­fluorene planes and the phenyl ring are 80.49 (6) and 76.57 (7)°. The crystal packing features O—H⋯N and O—H⋯O hydrogen bonds involving the bridging solvent water mol­ecules, which link the mol­ecules into a three-dimensional network.

## Related literature

For the synthesis of the title compound, see: Wong *et al.* (2001 ▶). 
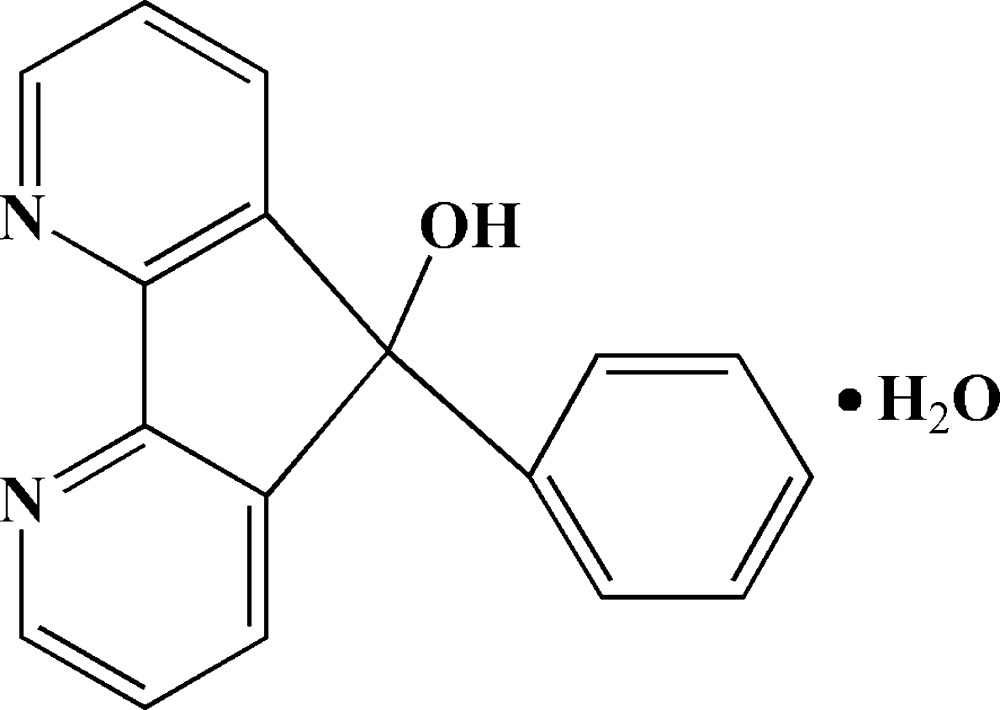


## Experimental

### 

#### Crystal data

C_17_H_12_N_2_O·H_2_O
*M* *_r_* = 278.30
Triclinic, 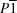

*a* = 8.8703 (13) Å
*b* = 9.1691 (18) Å
*c* = 18.643 (3) Å
α = 86.745 (14)°
β = 86.943 (12)°
γ = 67.798 (16)°
*V* = 1400.8 (4) Å^3^
*Z* = 4
Cu *K*α radiation
μ = 0.71 mm^−1^
*T* = 291 K
0.24 × 0.22 × 0.20 mm

#### Data collection

Bruker SMART CCD area-detector diffractometer
Absorption correction: multi-scan (*SADABS*; Sheldrick, 2004 ▶) *T*_min_ = 0.848, *T*_max_ = 0.871
10727 measured reflections
4893 independent reflections
3755 reflections with *I* > 2σ(*I*)
*R*_int_ = 0.034

#### Refinement

*R*[*F* ^2^ > 2σ(*F* ^2^)] = 0.052
*wR*(*F* ^2^) = 0.151
*S* = 1.04
4893 reflections
375 parameters
H-atom parameters constrained
Δρ_max_ = 0.63 e Å^−3^
Δρ_min_ = −0.37 e Å^−3^

### 

Data collection: *SMART* (Bruker, 2007 ▶); cell refinement: *SAINT* (Bruker, 2007 ▶); data reduction: *SAINT*; program(s) used to solve structure: *SHELXS97* (Sheldrick, 2008 ▶); program(s) used to refine structure: *SHELXL97* (Sheldrick, 2008 ▶); molecular graphics: *SHELXTL* (Sheldrick, 2008 ▶); software used to prepare material for publication: *SHELXTL*.

## Supplementary Material

Crystal structure: contains datablock(s) global, I. DOI: 10.1107/S1600536812021940/ez2292sup1.cif

Structure factors: contains datablock(s) I. DOI: 10.1107/S1600536812021940/ez2292Isup2.hkl

Supplementary material file. DOI: 10.1107/S1600536812021940/ez2292Isup3.cml

Additional supplementary materials:  crystallographic information; 3D view; checkCIF report

## Figures and Tables

**Table 1:** Hydrogen-bond geometry (Å, °)

*D*—H⋯*A*	*D*—H	H⋯*A*	*D*⋯*A*	*D*—H⋯*A*
O1—H1*A*⋯O2	0.82	1.85	2.668 (2)	173
O3—H3*A*⋯O4^i^	0.82	1.89	2.705 (2)	174
O4—H4*B*⋯N3^ii^	0.85	2.01	2.839 (2)	164
O2—H2*B*⋯N2^iii^	0.85	1.91	2.749 (2)	171

Symmetry codes: (i) 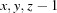; (ii) 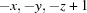; (iii) 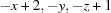.

**Fig. 1.**
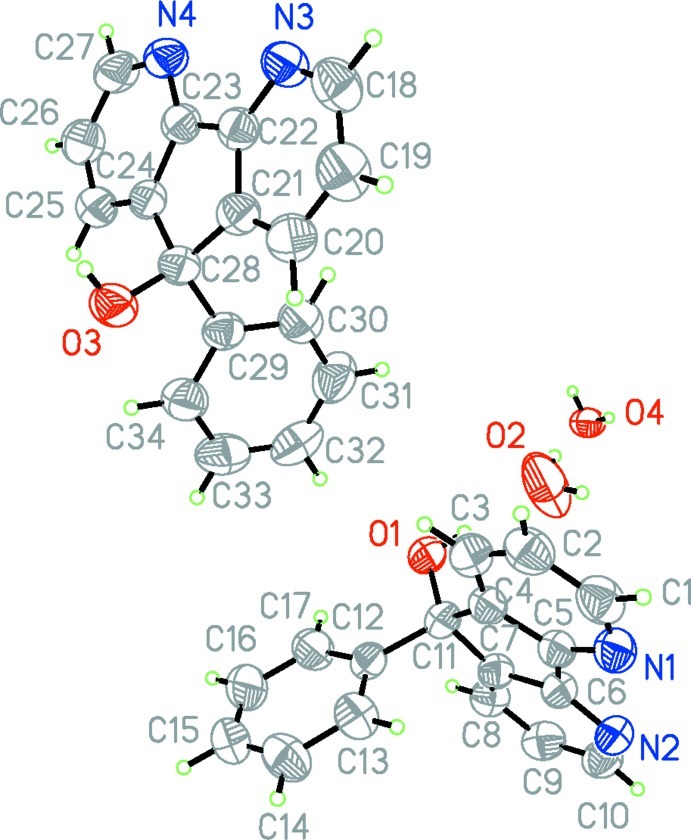
The molecule of the title compound, with displacement ellipsoids for non-H atoms drawn at the 30% probability level.

**Fig. 2.**
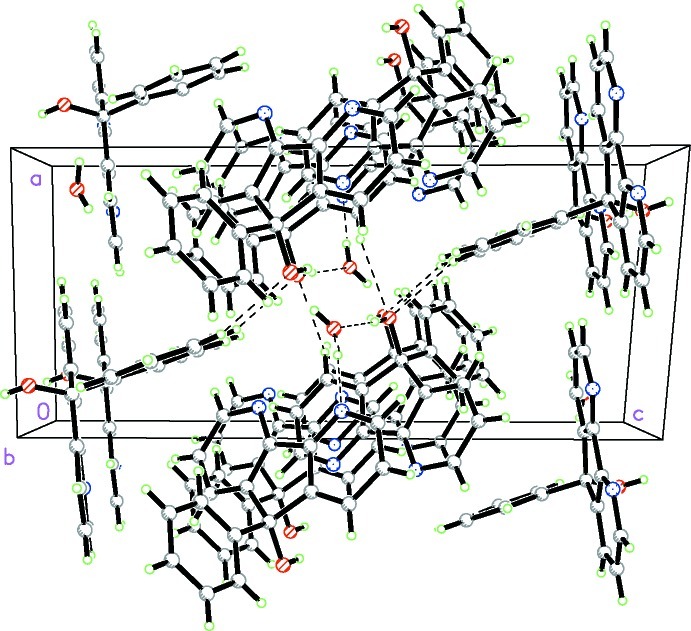
A packing diagram of the title compound, viewed down the b axis, with hydrogen bonding shown as dashed lines.

**Fig. 3.**
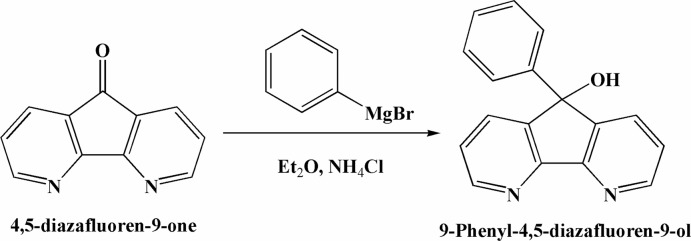
The synthetic route to the title compound.

## supplementary crystallographic information

### Comment

The title compound, containing two N atoms and a hydroxyl group, is a valuable intermediate in the preparation of medicinal compounds and an important ligand for the synthesis of functional metal-organic frameworks (MOFS). It was synthesized utilising a Grignard reagent to produce the target compound according to a literature reaction (Wong *et al.*, 2001).

The title compound crystallizes with two molecules in the asymmetric unit, as shown in Fig. 1. Moreover, the 4,5-diazafluorene fragment is approximately planar, with r.m.s. deviations of 0.0448 Å and 0.0198 Å, respectively, and the dihedral angle between the 4,5-diazafluorene plane and the phenyl fragment are 80.49 (6)° and 76.57 (7)°. As shown in Fig. 2, due to the existence of the bridging solvent water molecules the crystal packing is stabilized through O—H···N and O—H···O hydrogen bonds, which link the molecules into a three-dimensional network.

### Experimental

Reagents and solvents were of commercially available quality and the synthetic route (Wong *et al.*, 2001) to the title compound is shown in Fig. 3. The Grignard reagents were prepared from magnesium powder (0.486 g, 20 mmol) in Et_2_O (5 ml) and the corresponding arylbromide (20 mmol) in Et_2_O (15 ml). The cooled Grignard solution was diluted with dry Et_2_O (20 ml) and 4,5-diazafluoren-9-one (1.82 g, 10 mmol) was added to the Grignard solution. The mixture was refluxed for 2–6 h and then stirred for another 2 h at room temperature. The reaction mixture, was hydrolyzed with saturated NH_4_Cl solution, extracted with Et_2_O, washed with brine, dried over MgSO_4_. Evaporation of the solvent under reduced pressure yielded the crude product. Purification was effected by column chromatography (SiO_2_, EtOAc/Hexane = 1/5) and recrystallization from CH_2_Cl_2_ and hexane yielded colorless crystal.

### Refinement

Carbon-bound H-atoms were placed in calculated positions (C—H = 0.93–0.97 Å) and were included in the refinement in the riding model approximation, with *U*_iso_(H) set to 1.2*U*_eq_(C). The water H-atoms were located in a difference Fourier map, but were refined utilising the riding model with O—H = 0.85 Å and *U*_iso_(H) = 1.2*U*_eq_(O). The highest difference density of 0.629 e Å^-3^ is 1.20 Å from O2.

### Crystal data

**Table:**

C_17_H_12_N_2_O·H_2_O	*Z* = 4
*M**_r_* = 278.30	*F*(000) = 584
Triclinic, *P*1	*D*_x_ = 1.320 Mg m^−^^3^
*a* = 8.8703 (13) Å	Cu *K*α radiation, λ = 1.54184 Å
*b* = 9.1691 (18) Å	θ = 0.9–0.9°
*c* = 18.643 (3) Å	µ = 0.71 mm^−^^1^
α = 86.745 (14)°	*T* = 291 K
β = 86.943 (12)°	Prismatic, colorless
γ = 67.798 (16)°	0.24 × 0.22 × 0.20 mm
*V* = 1400.8 (4) Å^3^	

### Data collection

**Table:**

Bruker SMART CCD area-detector diffractometer	4893 independent reflections
Radiation source: fine-focus sealed tube	3755 reflections with *I* > 2σ(*I*)
Graphite monochromator	*R*_int_ = 0.034
phi and ω scans	θ_max_ = 66.6°, θ_min_ = 4.8°
Absorption correction: multi-scan (*SADABS*; Sheldrick, 2004)	*h* = −10→10
*T*_min_ = 0.848, *T*_max_ = 0.871	*k* = −10→9
10727 measured reflections	*l* = −22→20

### Refinement

**Table:**

Refinement on *F*^2^	Primary atom site location: structure-invariant direct methods
Least-squares matrix: full	Secondary atom site location: difference Fourier map
*R*[*F*^2^ > 2σ(*F*^2^)] = 0.052	Hydrogen site location: inferred from neighbouring sites
*wR*(*F*^2^) = 0.151	H-atom parameters constrained
*S* = 1.04	*w* = 1/[σ^2^(*F*_o_^2^) + (0.0787*P*)^2^ + 0.165*P*] where *P* = (*F*_o_^2^ + 2*F*_c_^2^)/3
4893 reflections	(Δ/σ)_max_ < 0.001
375 parameters	Δρ_max_ = 0.63 e Å^−^^3^
0 restraints	Δρ_min_ = −0.37 e Å^−^^3^

### Special details

**Table:**

Geometry. All e.s.d.'s (except the e.s.d. in the dihedral angle between two l.s. planes) are estimated using the full covariance matrix. The cell e.s.d.'s are taken into account individually in the estimation of e.s.d.'s in distances, angles and torsion angles; correlations between e.s.d.'s in cell parameters are only used when they are defined by crystal symmetry. An approximate (isotropic) treatment of cell e.s.d.'s is used for estimating e.s.d.'s involving l.s. planes.
Refinement. Refinement of *F*^2^ against ALL reflections. The weighted *R*-factor *wR* and goodness of fit *S* are based on *F*^2^, conventional *R*-factors *R* are based on *F*, with *F* set to zero for negative *F*^2^. The threshold expression of *F*^2^ > σ(*F*^2^) is used only for calculating *R*-factors(gt) *etc*. and is not relevant to the choice of reflections for refinement. *R*-factors based on *F*^2^ are statistically about twice as large as those based on *F*, and *R*- factors based on ALL data will be even larger.

### Fractional atomic coordinates and isotropic or equivalent isotropic displacement parameters (Å^2^)

**Table:**

	*x*	*y*	*z*	*U*_iso_*/*U*_eq_	
O1	0.57816 (17)	0.33453 (18)	0.42450 (9)	0.0548 (4)	
H1A	0.5908	0.2525	0.4479	0.082*	
O3	0.2007 (2)	0.3172 (2)	0.04084 (9)	0.0590 (4)	
H3A	0.1708	0.2943	0.0037	0.089*	
N4	−0.1757 (2)	0.0898 (2)	0.10802 (11)	0.0527 (4)	
N1	1.1101 (2)	−0.0146 (2)	0.37662 (12)	0.0560 (5)	
C12	0.7024 (2)	0.4852 (2)	0.35363 (12)	0.0469 (5)	
N2	1.1083 (2)	0.1802 (2)	0.50497 (11)	0.0550 (5)	
C6	0.9881 (2)	0.2098 (2)	0.45896 (12)	0.0470 (5)	
C5	0.9891 (2)	0.1169 (2)	0.39702 (12)	0.0465 (5)	
N3	0.1634 (2)	−0.1697 (2)	0.07988 (11)	0.0535 (4)	
C22	0.1298 (2)	−0.0174 (2)	0.08946 (11)	0.0461 (5)	
C11	0.7318 (2)	0.3365 (2)	0.40160 (12)	0.0466 (5)	
C4	0.8405 (2)	0.1867 (2)	0.36401 (12)	0.0477 (5)	
C24	−0.0120 (2)	0.2490 (2)	0.10714 (11)	0.0452 (4)	
C23	−0.0324 (2)	0.1074 (2)	0.10219 (11)	0.0451 (4)	
C7	0.8388 (2)	0.3341 (2)	0.46360 (11)	0.0453 (4)	
C28	0.1679 (2)	0.2274 (2)	0.09947 (12)	0.0474 (5)	
C27	−0.3054 (3)	0.2230 (3)	0.11991 (14)	0.0596 (6)	
H27	−0.4074	0.2162	0.1250	0.071*	
C21	0.2462 (2)	0.0511 (2)	0.08844 (11)	0.0469 (5)	
C25	−0.1472 (3)	0.3845 (3)	0.11780 (14)	0.0561 (5)	
H25	−0.1385	0.4820	0.1201	0.067*	
C29	0.2253 (2)	0.2752 (2)	0.16648 (12)	0.0476 (5)	
C10	1.0747 (3)	0.2791 (3)	0.55871 (13)	0.0596 (6)	
H10	1.1552	0.2628	0.5917	0.072*	
C3	0.8106 (3)	0.1166 (3)	0.30651 (13)	0.0575 (5)	
H3	0.7123	0.1595	0.2832	0.069*	
C17	0.5615 (3)	0.6152 (3)	0.36287 (18)	0.0709 (7)	
H17	0.4828	0.6115	0.3972	0.085*	
C1	1.0770 (3)	−0.0803 (3)	0.32010 (15)	0.0611 (6)	
H1	1.1571	−0.1726	0.3038	0.073*	
C30	0.2676 (3)	0.1754 (3)	0.22721 (15)	0.0665 (6)	
H30	0.2599	0.0770	0.2274	0.080*	
C20	0.4076 (3)	−0.0435 (3)	0.07687 (14)	0.0579 (6)	
H20	0.4888	−0.0024	0.0767	0.069*	
C13	0.8185 (3)	0.4965 (3)	0.30300 (13)	0.0585 (5)	
H13	0.9151	0.4098	0.2965	0.070*	
C9	0.9288 (3)	0.4035 (3)	0.56844 (13)	0.0607 (6)	
H9	0.9124	0.4673	0.6075	0.073*	
C26	−0.2968 (3)	0.3690 (3)	0.12501 (15)	0.0625 (6)	
H26	−0.3911	0.4569	0.1333	0.075*	
C8	0.8065 (3)	0.4337 (3)	0.52010 (13)	0.0539 (5)	
H8	0.7070	0.5173	0.5254	0.065*	
C34	0.2348 (3)	0.4217 (3)	0.16785 (15)	0.0620 (6)	
H34	0.2041	0.4912	0.1282	0.074*	
C16	0.5362 (3)	0.7527 (3)	0.3209 (2)	0.0881 (10)	
H16	0.4397	0.8395	0.3272	0.106*	
C2	0.9330 (3)	−0.0211 (3)	0.28446 (14)	0.0623 (6)	
H2	0.9180	−0.0732	0.2457	0.075*	
C32	0.3354 (3)	0.3644 (4)	0.28715 (16)	0.0728 (8)	
H32	0.3758	0.3932	0.3268	0.087*	
C31	0.3208 (4)	0.2207 (4)	0.28722 (16)	0.0765 (8)	
H31	0.3469	0.1536	0.3279	0.092*	
C18	0.3210 (3)	−0.2570 (3)	0.06740 (15)	0.0625 (6)	
H18	0.3487	−0.3634	0.0594	0.075*	
C14	0.7941 (4)	0.6334 (3)	0.26199 (14)	0.0682 (7)	
H14	0.8739	0.6386	0.2287	0.082*	
C33	0.2895 (4)	0.4657 (3)	0.22770 (18)	0.0739 (7)	
H33	0.2956	0.5647	0.2280	0.089*	
C19	0.4451 (3)	−0.2013 (3)	0.06554 (16)	0.0674 (7)	
H19	0.5525	−0.2686	0.0568	0.081*	
C15	0.65056 (16)	0.76264 (14)	0.27072 (8)	0.0752 (8)	
H15	0.6320	0.8548	0.2429	0.090*	
O4	0.12630 (16)	0.23352 (14)	0.91460 (8)	0.0639 (4)	
H4A	0.1958	0.1556	0.8933	0.077*	
H4B	0.0329	0.2298	0.9103	0.077*	
O2	0.61191 (16)	0.08322 (14)	0.51118 (8)	0.1338 (13)	
H2A	0.5490	0.0608	0.5420	0.161*	
H2B	0.7032	0.0086	0.5039	0.161*	

### Atomic displacement parameters (Å^2^)

**Table:**

	*U*^11^	*U*^22^	*U*^33^	*U*^12^	*U*^13^	*U*^23^
O1	0.0393 (7)	0.0561 (8)	0.0680 (10)	−0.0176 (6)	−0.0035 (7)	0.0057 (7)
O3	0.0694 (10)	0.0672 (10)	0.0527 (9)	−0.0409 (8)	−0.0022 (8)	0.0071 (7)
N4	0.0444 (9)	0.0561 (10)	0.0614 (11)	−0.0231 (8)	−0.0028 (8)	−0.0020 (8)
N1	0.0481 (9)	0.0473 (9)	0.0674 (12)	−0.0126 (8)	0.0029 (8)	−0.0026 (9)
C12	0.0439 (10)	0.0470 (10)	0.0501 (11)	−0.0167 (8)	−0.0089 (8)	−0.0005 (9)
N2	0.0476 (9)	0.0579 (10)	0.0601 (11)	−0.0207 (8)	−0.0108 (8)	0.0064 (9)
C6	0.0425 (10)	0.0480 (10)	0.0508 (11)	−0.0183 (8)	−0.0047 (8)	0.0058 (9)
C5	0.0424 (10)	0.0432 (10)	0.0535 (12)	−0.0163 (8)	−0.0005 (8)	0.0030 (9)
N3	0.0522 (10)	0.0471 (9)	0.0624 (11)	−0.0207 (8)	0.0016 (8)	−0.0012 (8)
C22	0.0451 (10)	0.0487 (11)	0.0449 (11)	−0.0187 (9)	−0.0006 (8)	0.0019 (8)
C11	0.0377 (9)	0.0475 (10)	0.0526 (12)	−0.0136 (8)	−0.0040 (8)	−0.0006 (9)
C4	0.0460 (10)	0.0446 (10)	0.0527 (12)	−0.0178 (8)	−0.0004 (9)	0.0011 (9)
C24	0.0442 (10)	0.0497 (11)	0.0440 (10)	−0.0203 (8)	−0.0068 (8)	0.0047 (8)
C23	0.0445 (10)	0.0505 (11)	0.0425 (10)	−0.0207 (8)	−0.0029 (8)	0.0020 (8)
C7	0.0412 (10)	0.0481 (10)	0.0468 (11)	−0.0176 (8)	−0.0009 (8)	0.0022 (8)
C28	0.0463 (10)	0.0510 (11)	0.0500 (11)	−0.0245 (9)	−0.0021 (8)	0.0027 (9)
C27	0.0399 (10)	0.0682 (14)	0.0724 (16)	−0.0220 (10)	−0.0043 (10)	−0.0030 (12)
C21	0.0462 (10)	0.0528 (11)	0.0443 (11)	−0.0219 (9)	0.0009 (8)	−0.0003 (9)
C25	0.0556 (12)	0.0486 (11)	0.0644 (14)	−0.0194 (10)	−0.0081 (10)	−0.0005 (10)
C29	0.0393 (9)	0.0532 (11)	0.0536 (12)	−0.0213 (8)	0.0005 (8)	−0.0023 (9)
C10	0.0610 (13)	0.0718 (15)	0.0536 (13)	−0.0333 (12)	−0.0128 (10)	0.0058 (11)
C3	0.0611 (13)	0.0555 (12)	0.0587 (14)	−0.0242 (10)	−0.0104 (10)	−0.0001 (10)
C17	0.0443 (11)	0.0556 (13)	0.106 (2)	−0.0134 (10)	0.0038 (12)	0.0110 (13)
C1	0.0630 (13)	0.0463 (11)	0.0705 (15)	−0.0170 (10)	0.0095 (11)	−0.0099 (11)
C30	0.0804 (17)	0.0634 (14)	0.0623 (15)	−0.0338 (13)	−0.0138 (13)	0.0038 (12)
C20	0.0456 (11)	0.0644 (13)	0.0646 (14)	−0.0232 (10)	0.0043 (10)	0.0019 (11)
C13	0.0656 (13)	0.0520 (12)	0.0546 (13)	−0.0190 (10)	0.0051 (10)	−0.0052 (10)
C9	0.0651 (14)	0.0745 (15)	0.0495 (12)	−0.0338 (12)	−0.0007 (10)	−0.0061 (11)
C26	0.0443 (11)	0.0578 (13)	0.0785 (17)	−0.0110 (10)	−0.0050 (11)	−0.0018 (12)
C8	0.0513 (11)	0.0561 (12)	0.0533 (12)	−0.0195 (9)	0.0040 (9)	−0.0054 (10)
C34	0.0651 (14)	0.0554 (13)	0.0695 (15)	−0.0269 (11)	−0.0035 (12)	−0.0042 (11)
C16	0.0540 (14)	0.0560 (14)	0.143 (3)	−0.0101 (12)	−0.0166 (17)	0.0245 (17)
C2	0.0764 (16)	0.0549 (13)	0.0598 (14)	−0.0290 (12)	0.0008 (12)	−0.0085 (11)
C32	0.0537 (13)	0.094 (2)	0.0728 (17)	−0.0255 (13)	−0.0082 (12)	−0.0264 (15)
C31	0.0817 (18)	0.0847 (19)	0.0600 (16)	−0.0257 (15)	−0.0219 (14)	0.0006 (14)
C18	0.0596 (13)	0.0492 (12)	0.0726 (16)	−0.0151 (10)	0.0071 (11)	−0.0011 (11)
C14	0.0915 (19)	0.0644 (15)	0.0554 (14)	−0.0379 (14)	0.0036 (13)	−0.0015 (11)
C33	0.0756 (16)	0.0710 (16)	0.086 (2)	−0.0376 (14)	−0.0002 (14)	−0.0241 (15)
C19	0.0484 (12)	0.0633 (14)	0.0816 (18)	−0.0131 (11)	0.0123 (11)	−0.0009 (12)
C15	0.0812 (18)	0.0612 (15)	0.087 (2)	−0.0315 (13)	−0.0240 (15)	0.0240 (14)
O4	0.0665 (10)	0.0683 (10)	0.0674 (11)	−0.0373 (8)	−0.0043 (8)	−0.0011 (8)
O2	0.0964 (17)	0.0766 (14)	0.195 (3)	−0.0078 (13)	0.0435 (19)	0.0445 (17)

### Geometric parameters (Å, º)

**Table:**

O1—C11	1.413 (2)	C10—H10	0.9300
O1—H1A	0.8200	C3—C2	1.385 (4)
O3—C28	1.415 (3)	C3—H3	0.9300
O3—H3A	0.8200	C17—C16	1.394 (4)
N4—C23	1.339 (3)	C17—H17	0.9300
N4—C27	1.343 (3)	C1—C2	1.376 (4)
N1—C5	1.334 (3)	C1—H1	0.9300
N1—C1	1.341 (3)	C30—C31	1.380 (4)
C12—C17	1.374 (3)	C30—H30	0.9300
C12—C13	1.388 (3)	C20—C19	1.383 (4)
C12—C11	1.531 (3)	C20—H20	0.9300
N2—C10	1.335 (3)	C13—C14	1.382 (4)
N2—C6	1.341 (3)	C13—H13	0.9300
C6—C7	1.385 (3)	C9—C8	1.384 (3)
C6—C5	1.471 (3)	C9—H9	0.9300
C5—C4	1.389 (3)	C26—H26	0.9300
N3—C22	1.334 (3)	C8—H8	0.9300
N3—C18	1.337 (3)	C34—C33	1.378 (4)
C22—C21	1.396 (3)	C34—H34	0.9300
C22—C23	1.478 (3)	C16—C15	1.368 (4)
C11—C7	1.528 (3)	C16—H16	0.9300
C11—C4	1.531 (3)	C2—H2	0.9300
C4—C3	1.368 (3)	C32—C31	1.372 (4)
C24—C25	1.377 (3)	C32—C33	1.379 (4)
C24—C23	1.386 (3)	C32—H32	0.9300
C24—C28	1.532 (3)	C31—H31	0.9300
C7—C8	1.380 (3)	C18—C19	1.377 (4)
C28—C29	1.520 (3)	C18—H18	0.9300
C28—C21	1.521 (3)	C14—C15	1.382 (3)
C27—C26	1.378 (3)	C14—H14	0.9300
C27—H27	0.9300	C33—H33	0.9300
C21—C20	1.377 (3)	C19—H19	0.9300
C25—C26	1.385 (3)	C15—H15	0.9300
C25—H25	0.9300	O4—H4A	0.8499
C29—C34	1.379 (3)	O4—H4B	0.8499
C29—C30	1.389 (3)	O2—H2A	0.8500
C10—C9	1.376 (4)	O2—H2B	0.8500
			
C11—O1—H1A	109.5	C4—C3—H3	121.5
C28—O3—H3A	109.5	C2—C3—H3	121.5
C23—N4—C27	114.86 (19)	C12—C17—C16	120.1 (3)
C5—N1—C1	114.2 (2)	C12—C17—H17	119.9
C17—C12—C13	118.2 (2)	C16—C17—H17	119.9
C17—C12—C11	119.8 (2)	N1—C1—C2	124.7 (2)
C13—C12—C11	121.89 (19)	N1—C1—H1	117.6
C10—N2—C6	115.3 (2)	C2—C1—H1	117.6
N2—C6—C7	124.5 (2)	C31—C30—C29	120.7 (2)
N2—C6—C5	126.85 (19)	C31—C30—H30	119.6
C7—C6—C5	108.60 (18)	C29—C30—H30	119.6
N1—C5—C4	125.3 (2)	C21—C20—C19	118.0 (2)
N1—C5—C6	126.14 (19)	C21—C20—H20	121.0
C4—C5—C6	108.49 (18)	C19—C20—H20	121.0
C22—N3—C18	115.15 (19)	C14—C13—C12	121.6 (2)
N3—C22—C21	124.5 (2)	C14—C13—H13	119.2
N3—C22—C23	127.28 (19)	C12—C13—H13	119.2
C21—C22—C23	108.21 (18)	C10—C9—C8	119.9 (2)
O1—C11—C7	113.44 (18)	C10—C9—H9	120.0
O1—C11—C12	107.74 (16)	C8—C9—H9	120.0
C7—C11—C12	109.78 (17)	C27—C26—C25	119.8 (2)
O1—C11—C4	113.06 (17)	C27—C26—H26	120.1
C7—C11—C4	100.59 (16)	C25—C26—H26	120.1
C12—C11—C4	112.20 (18)	C7—C8—C9	117.0 (2)
C3—C4—C5	119.0 (2)	C7—C8—H8	121.5
C3—C4—C11	130.0 (2)	C9—C8—H8	121.5
C5—C4—C11	110.99 (19)	C33—C34—C29	120.4 (3)
C25—C24—C23	119.08 (19)	C33—C34—H34	119.8
C25—C24—C28	129.28 (19)	C29—C34—H34	119.8
C23—C24—C28	111.64 (18)	C15—C16—C17	121.3 (2)
N4—C23—C24	124.93 (19)	C15—C16—H16	119.3
N4—C23—C22	126.92 (19)	C17—C16—H16	119.3
C24—C23—C22	108.14 (17)	C1—C2—C3	119.7 (2)
C8—C7—C6	119.0 (2)	C1—C2—H2	120.2
C8—C7—C11	129.77 (19)	C3—C2—H2	120.2
C6—C7—C11	111.19 (18)	C31—C32—C33	119.3 (2)
O3—C28—C29	106.99 (16)	C31—C32—H32	120.4
O3—C28—C21	112.36 (18)	C33—C32—H32	120.4
C29—C28—C21	112.57 (18)	C32—C31—C30	120.2 (3)
O3—C28—C24	112.88 (17)	C32—C31—H31	119.9
C29—C28—C24	111.63 (17)	C30—C31—H31	119.9
C21—C28—C24	100.50 (16)	N3—C18—C19	124.9 (2)
N4—C27—C26	124.2 (2)	N3—C18—H18	117.5
N4—C27—H27	117.9	C19—C18—H18	117.5
C26—C27—H27	117.9	C13—C14—C15	119.8 (2)
C20—C21—C22	118.6 (2)	C13—C14—H14	120.1
C20—C21—C28	129.95 (19)	C15—C14—H14	120.1
C22—C21—C28	111.49 (18)	C34—C33—C32	120.7 (3)
C24—C25—C26	117.1 (2)	C34—C33—H33	119.7
C24—C25—H25	121.5	C32—C33—H33	119.7
C26—C25—H25	121.5	C18—C19—C20	118.9 (2)
C34—C29—C30	118.6 (2)	C18—C19—H19	120.6
C34—C29—C28	119.7 (2)	C20—C19—H19	120.6
C30—C29—C28	121.70 (19)	C16—C15—C14	118.94 (18)
N2—C10—C9	124.2 (2)	C16—C15—H15	120.5
N2—C10—H10	117.9	C14—C15—H15	120.5
C9—C10—H10	117.9	H4A—O4—H4B	107.7
C4—C3—C2	117.1 (2)	H2A—O2—H2B	114.5
			
C10—N2—C6—C7	1.2 (3)	C23—N4—C27—C26	0.9 (4)
C10—N2—C6—C5	−177.4 (2)	N3—C22—C21—C20	−0.1 (3)
C1—N1—C5—C4	−1.1 (3)	C23—C22—C21—C20	−179.6 (2)
C1—N1—C5—C6	177.1 (2)	N3—C22—C21—C28	178.8 (2)
N2—C6—C5—N1	0.9 (3)	C23—C22—C21—C28	−0.8 (2)
C7—C6—C5—N1	−177.8 (2)	O3—C28—C21—C20	58.5 (3)
N2—C6—C5—C4	179.4 (2)	C29—C28—C21—C20	−62.3 (3)
C7—C6—C5—C4	0.6 (2)	C24—C28—C21—C20	178.8 (2)
C18—N3—C22—C21	−1.2 (3)	O3—C28—C21—C22	−120.1 (2)
C18—N3—C22—C23	178.3 (2)	C29—C28—C21—C22	118.99 (19)
C17—C12—C11—O1	26.8 (3)	C24—C28—C21—C22	0.1 (2)
C13—C12—C11—O1	−156.7 (2)	C23—C24—C25—C26	1.8 (3)
C17—C12—C11—C7	−97.1 (2)	C28—C24—C25—C26	−178.1 (2)
C13—C12—C11—C7	79.3 (3)	O3—C28—C29—C34	24.7 (3)
C17—C12—C11—C4	151.9 (2)	C21—C28—C29—C34	148.6 (2)
C13—C12—C11—C4	−31.7 (3)	C24—C28—C29—C34	−99.2 (2)
N1—C5—C4—C3	1.2 (3)	O3—C28—C29—C30	−155.8 (2)
C6—C5—C4—C3	−177.3 (2)	C21—C28—C29—C30	−31.9 (3)
N1—C5—C4—C11	−179.7 (2)	C24—C28—C29—C30	80.2 (3)
C6—C5—C4—C11	1.9 (2)	C6—N2—C10—C9	0.2 (3)
O1—C11—C4—C3	54.4 (3)	C5—C4—C3—C2	−0.4 (3)
C7—C11—C4—C3	175.7 (2)	C11—C4—C3—C2	−179.3 (2)
C12—C11—C4—C3	−67.7 (3)	C13—C12—C17—C16	1.4 (4)
O1—C11—C4—C5	−124.57 (19)	C11—C12—C17—C16	177.9 (3)
C7—C11—C4—C5	−3.3 (2)	C5—N1—C1—C2	0.3 (4)
C12—C11—C4—C5	113.3 (2)	C34—C29—C30—C31	−1.1 (4)
C27—N4—C23—C24	−0.4 (3)	C28—C29—C30—C31	179.4 (2)
C27—N4—C23—C22	179.7 (2)	C22—C21—C20—C19	1.2 (3)
C25—C24—C23—N4	−1.0 (3)	C28—C21—C20—C19	−177.4 (2)
C28—C24—C23—N4	178.9 (2)	C17—C12—C13—C14	−0.7 (4)
C25—C24—C23—C22	178.90 (19)	C11—C12—C13—C14	−177.2 (2)
C28—C24—C23—C22	−1.2 (2)	N2—C10—C9—C8	−1.0 (4)
N3—C22—C23—N4	1.6 (4)	N4—C27—C26—C25	−0.1 (4)
C21—C22—C23—N4	−178.9 (2)	C24—C25—C26—C27	−1.3 (4)
N3—C22—C23—C24	−178.3 (2)	C6—C7—C8—C9	1.1 (3)
C21—C22—C23—C24	1.2 (2)	C11—C7—C8—C9	−179.3 (2)
N2—C6—C7—C8	−1.9 (3)	C10—C9—C8—C7	0.2 (3)
C5—C6—C7—C8	176.88 (19)	C30—C29—C34—C33	1.8 (4)
N2—C6—C7—C11	178.37 (19)	C28—C29—C34—C33	−178.8 (2)
C5—C6—C7—C11	−2.8 (2)	C12—C17—C16—C15	−0.9 (5)
O1—C11—C7—C8	−55.0 (3)	N1—C1—C2—C3	0.4 (4)
C12—C11—C7—C8	65.6 (3)	C4—C3—C2—C1	−0.3 (4)
C4—C11—C7—C8	−176.0 (2)	C33—C32—C31—C30	2.8 (4)
O1—C11—C7—C6	124.70 (19)	C29—C30—C31—C32	−1.2 (5)
C12—C11—C7—C6	−114.7 (2)	C22—N3—C18—C19	1.4 (4)
C4—C11—C7—C6	3.7 (2)	C12—C13—C14—C15	−0.5 (4)
C25—C24—C28—O3	−59.5 (3)	C29—C34—C33—C32	−0.1 (4)
C23—C24—C28—O3	120.6 (2)	C31—C32—C33—C34	−2.2 (4)
C25—C24—C28—C29	61.0 (3)	N3—C18—C19—C20	−0.4 (4)
C23—C24—C28—C29	−118.9 (2)	C21—C20—C19—C18	−1.0 (4)
C25—C24—C28—C21	−179.4 (2)	C17—C16—C15—C14	−0.3 (4)
C23—C24—C28—C21	0.7 (2)	C13—C14—C15—C16	1.0 (4)

### Hydrogen-bond geometry (Å, º)

**Table:**

*D*—H···*A*	*D*—H	H···*A*	*D*···*A*	*D*—H···*A*
O1—H1*A*···O2	0.82	1.85	2.668 (2)	173
O3—H3*A*···O4^i^	0.82	1.89	2.705 (2)	174
O4—H4*B*···N3^ii^	0.85	2.01	2.839 (2)	164
O2—H2*B*···N2^iii^	0.85	1.91	2.749 (2)	171

Symmetry codes: (i) *x*, *y*, *z*−1; (ii) −*x*, −*y*, −*z*+1; (iii) −*x*+2, −*y*, −*z*+1.
